# Chronic exposure to PM2.5 aggravates SLE manifestations in lupus-prone mice

**DOI:** 10.1186/s12989-021-00407-0

**Published:** 2021-03-25

**Authors:** Victor Yuji Yariwake, Janaína Iannicelli Torres, Amandda Rakell Peixoto dos Santos, Sarah Cristina Ferreira Freitas, Kátia De Angelis, Sylvia Costa Lima Farhat, Niels Olsen Saraiva Câmara, Mariana Matera Veras

**Affiliations:** 1 Laboratory of Experimental Air Pollution, Department of Pathology, School of Medicine, University of São Paulo, Av. Dr. Arnaldo, 455 - 1st floor (room 1220), São Paulo, SP 01246-903 Brazil; 2 Laboratory of Experimental Cellular Immunology, Department of Medicine, Division of Nephrology, Federal University of São Paulo, São Paulo, Brazil; 3 Laboratory of Translational Physiology, Universidade Nove de Julho (UNINOVE), São Paulo, Brazil; 4 Pediatric Rheumatology Unit, Children’s Institute of Hospital das Clínicas, School of Medicine, University of São Paulo, São Paulo, Brazil; 5 Laboratory of Transplant Immunology, Department of Immunology, Institute of Biomedical Sciences, University of São Paulo, São Paulo, Brazil

**Keywords:** Air pollution, Systemic lupus erythematosus, Particulate matter, Lupus nephritis, Autoimmunity, Environmental exposure

## Abstract

**Background:**

Air pollution causes negative impacts on health. Systemic lupus erythematosus (SLE) is an autoimmune disease with diverse clinical manifestations and multifactorial etiology. Recent studies suggest that air pollution can trigger SLE and induce disease activity. However, this association has not been deeply investigated. Thus, the aim of this study was to evaluate whether exposure to fine particulate matter (PM2.5) exacerbates SLE manifestations, focusing on renal complications, in a lupus-prone animal model. Female NZBWF1 mice were exposed daily to 600 μg/m^3^ of inhaled concentrated ambient particles (CAP) or filtered air (FA). Survival rate, body weight, weight of organs (kidney, spleen, thymus, liver and heart), blood cell count, proteinuria, kidney stereology, renal histopathology, gene expression and oxidative stress were analyzed.

**Results:**

Female NZBW mice exposed to CAP showed decreased survival, increased circulating neutrophils, early onset of proteinuria and increased kidney weight with renal cortex enlargement when compared to NZBW mice exposed to FA.

**Conclusions:**

This work shows that air pollution aggravates some SLE manifestations in lupus-prone mice. These results reinforce the need of reducing air pollutant levels in order to promote a better quality of life for individuals diagnosed with SLE.

**Supplementary Information:**

The online version contains supplementary material available at 10.1186/s12989-021-00407-0.

## Background

Systemic lupus erythematosus (SLE) is a complex autoimmune disease with diverse clinical manifestations. Skin, joints, blood and kidneys are the most affected by chronic inflammatory processes [1, 2]. SLE etiology remains not fully elucidated. However, genetic and environmental factors contribute to their development [3, 4]. Variations in HLA-DR (recognition of self and non-self-antigens) and type-I interferon genes are commonly reported among SLE patients [5, 6]. However, these genetic variations do not explain the increase of SLE incidence worldwide over the last decades [7]. Among environmental factors, UV radiation, viral infections (Epistein-Barr), drugs (procainamide and hidralazine), alcohol, tobacco and silica are considered risk factors associated with SLE development [8, 9].

Recently, epidemiological evidence suggest that exposure to air pollution could be a potential contributor for SLE activation and aggravation through systemic inflammation and oxidative stress [10, 11]. Air pollution is the major environmental risk for health; last reports from the World Health Organization (WHO) showed that annually more than 4 million deaths could be associated with exposure to ambient air pollution [12, 13]. Moreover, epidemiological and experimental studies have demonstrated that exposures are associated with respiratory, cardiovascular, neurologic, endocrine and reproductive disorders [14–16].

Air pollutants comprise a mixture of gases, organic compounds, metals and particles [17]. Within this mixture, fine particulate matter (PM2.5) is the pollutant with the strongest relationship with negative health outcomes and mortality [18, 19]. The process of fossil fuel combustion is the main source of PM2.5 generation and its small size facilitates penetration deeply into the respiratory system. Inhalation of these particles are associated with the promotion of inflammatory processes and oxidative stress [20, 21].

Immunopathology of SLE comprises the production of autoantibodies, mainly against nuclear antigens, that form immune complexes (IC). These IC deposit in tissues and vessels amplifying inflammatory responses, characterized by complement activation, recruitment of immune cells and tissue damage [22, 23]. Kidney complications are very common in SLE due to deposition of IC in glomeruli. Some patients can develop proteinuria, glomerulosclerosis and kidney failure depending on the disease severity [22, 24]. Plausible mechanisms involved in the association of air pollution and lupus include: the systemic inflammatory response and oxidative stress triggered by particles in the lungs; alteration of Th1/Th2/Th17 cells ratio and activation of B cells and dendritic cells induced by the translocation of particles into the bloodstream; increased apoptosis and defective clearance of apoptotic debris stimulating autoimmunity; and epigenetic changes [10, 25–27].

Therefore, in this novel study we aimed to evaluate whether chronic exposure to PM2.5 exacerbates clinical manifestations of SLE (focusing on kidney and systemic involvement) on female mice spontaneously predisposed to SLE development (NZBWF1 strain). We investigated a murine model that mimics the human condition to study disease progression in predisposed individuals for a better understanding of air pollution negative impacts on SLE.

## Methods

### Animals and experimental design

**Fig. 1:**
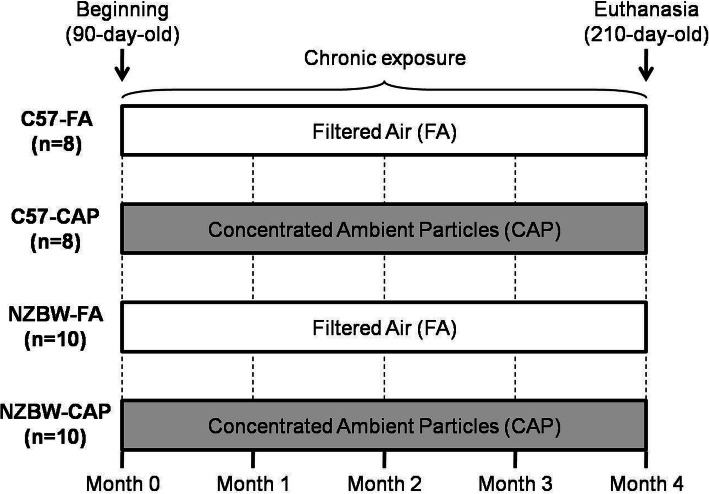
Experimental design. C57-FA: control mice exposed to filtered air; C57-CAP: control mice exposed to concentrated ambient particles (air pollution); NZBW-FA: lupus-prone mice exposed to filtered air; NZBW-CAP: lupus-prone mice exposed to concentrated ambient particles (air pollution)

Female NZBWF1 mice, 75-day-old, were purchased from Jackson Laboratory (Bar Harbor, USA) and utilized as SLE model (*n* = 20). Female C57BL/6 mice of the same age were obtained from University of Campinas and utilized as controls (*n* = 16). After 15 days of acclimatization, each strain was randomly subdivided into two groups, one exposed to filtered air (FA) and one exposed to concentrated ambient particles (CAP). In total, animals were divided into four groups: control mice exposed to FA (C57-FA) (*n* = 8); control mice exposed to CAP (C57-CAP) (n = 8); lupus-prone mice exposed to FA (NZBW-FA) (*n* = 10); lupus-prone mice exposed to CAP (NZBW-CAP) (n = 10). Exposure to FA or CAP occurred for four months (starting from 90-day-old until 210-day-old). During the exposure period, animals were weighed, and urine samples were collected monthly. Also, the health status of the animals was checked daily, and survival was evaluated. Experimental design is shown in Fig. 1. Animals were allocated in cages (4–5 animals/cage) lined with pine wood shavings, and cardboard tubes and cotton balls for nesting were used as environmental enrichment strategies. Food and water were provided ad libitum. Except during CAP exposures, cages were put into closed ventilated racks supplied with HEPA filtered air, controlled temperature of 21–23 °C and under light/dark cycle of 12 h/12 h. Animal procedures were approved by the Ethical Committee for Animal Research of the School of Medicine of University of São Paulo, under protocol number 095/17.

### Daily exposure to PM2.5

Animal exposure to PM2.5 was performed in the Harvard Ambient Particle Concentrator (HAPC) [28] (Additional file 1). The HAPC is located on the campus of the School of Medicine of São Paulo University, close to a high-traffic road (23°33′18.1″S 46°40′15.0″W). The HAPC enriches the concentration of ambient particles by a factor of nearly 17 times the ambient levels of PM2.5, without modifying its physicochemical properties. A DataRam DR-4000 (Thermo Fisher Scientific, USA) was used to monitor the concentration of PM2.5 during the exposure. The dose of exposure is controlled by increasing or decreasing the daily exposure time period in order to give to the animals the same dose every day (600 μg/m^3^). This concentration represents the adjusted air concentration that São Paulo residents are exposed to [29]. This adjusted air concentration (C_air-adj_) of 600 μg/m^3^ in 1 day/24hs was determined following current methodology of Environmental Protection Agency (EPA) [30], as follows:

$$ {\mathrm{C}}_{\mathrm{air}-\mathrm{adj}}={\mathrm{C}}_{\mathrm{air}}\ \mathrm{x}\ \mathrm{ET}\ \mathrm{x}\ \mathrm{EF}\ \mathrm{x}\ \mathrm{ED}/\mathrm{AT}\ \mathrm{x}\ 1\mathrm{day}/24\ \mathrm{hours} $$

Where:

- C_air_ = Concentration of contaminant in air (mg/m^3^) = 25 μg/m^3^ (São Paulo)
- ET = Exposure time (hours/day) = 24 h/day
- EF = Exposure frequency (days/year) = 365 days/year
- ED = Exposure duration (years) = 1 year
- AT = Averaging time (days) = 365 days

Before the initiation of the exposures, animals were also acclimatized to the HAPC for 5 days to reduce the distress of handling. Exposures were performed in whole-body inhalation chambers (FA or CAP) as described in previous studies [29, 31, 32]. Briefly, both FA and CAP groups were exposed at the same time to the same conditions, except for the presence of concentrated particles in CAP chamber. The two exposure chambers are assembled side by side (see Additional file 1) and they work in same conditions of pressure (0.99 atm), air flow (10 L/min), temperature and noise; the difference is that FA chamber receives HEPA filtered air.

### PM2.5 characterization

Samples of ambient PM2.5 were collected for characterization of PM2.5 elemental composition. For this, polycarbonate filter samples were placed into Harvard Virtual Impactor (a device that permits only the passage of particles with diameter less than 2,5 μm) for 24hs. A flux of 10 L/min was generated by a vacuum pump for retention of ambient PM2.5 in sample filters. Characterization of PM2.5 elemental composition was performed by energy dispersive X-ray spectroscopy (EDX) as previously described [33]. 20 filter samples were analyzed by EDX.

### Proteinuria analysis

Isolated urine samples were collected monthly by stimulation of perineal area as previous described [34]. Protein/creatinine ratio (PCR) and albumin/creatinine ratio (ACR) of urine samples were assessed. Quantification of total proteinuria was performed using Sensiprot kit (Labtest Diagnosis, Brazil). Procedures were conducted according to the manufacturer. Samples were diluted at 1:5 and incubated with color reagent at 37 °C/5 min. Absorbance was read at wavelength 600 nm. Quantification of proteinuria (mg/dL) was determined based on the absorbance of the sample (A_sa_) and standard (A_st_) through the equation “[(A_sa_ ÷ A_st_) x 50] x 5”, where 50 corresponds to the protein standard concentration in mg/dL and 5 to the dilution factor. Quantification of creatininuria was performed following modified Jaffe method (Labtest Diagnosis, Brazil). Samples were diluted at 1:125 and incubated with a solution of 80% picric acid, 1% m/v + 20% sodium hydroxide, 10% m/v at 25 °C/20 min. Absorbance was read at wavelength 520 nm. Quantification of creatininuria (mg/dL) was determined based on the absorbance of the sample (A_sa_) and standard (A_st_) through the equation “[(A_sa_) ÷ (A_st_) x 5] x 25”, where 5 corresponds to the standard concentration in mg/dL and 25 to the dilution factor (1:125) corrected by the creatinine standard concentration (5 mg/dL). Albuminuria quantification was performed using electrophoresis gel. Firstly, urine samples were treated with laemmli buffer (5% β-mercaptoethanol) and heat (95 °C/5 min) for protein denaturation. Proteins were run on a 10% SDS-polyacrylamide electrophoresis gel and stained with Coomassie Blue R-259. After the staining, gels were immersed in a destain solution (7.5% acetic acid + 25% methanol + 67.5% distilled water) and gel images were obtained from an Amersham Imager 600 (GE Healthcare, USA). Quantification of albuminuria (μg/mL) was performed by ImageJ software (Media Cybernetics, USA). Briefly, stained albumin in the samples was observed in bands at 66.5 kDa and the pixels of each band were measured and compared to a standard band (corresponding to 125 μg/mL of BSA). Note that for each gel there was a standard band control. Then, albuminuria were normalized by their creatinine concentration previously measured in the respective urine samples.

### Euthanasia procedures

Animals were euthanized right after the fourth month of exposure (at 210 day-old) through overdose inhalation of the anesthetic isoflurane (Cristalia, Brazil). Blood samples were collected for hemogram and serologic analyses. Kidneys, spleen, liver, heart and thymus were collected, weighed and stored for histological and molecular analyses.

### Hemogram analysis

Complete blood count was performed for blood cells screening. Red blood cells, white blood cells (including total count and percentage of neutrophils, eosinophils, basophils, monocytes and lymphocytes) and platelets were counted. Analysis was conducted using the automated hematologic counter pocH 100iV (Sysmex, Brazil).

### Anti-DNA quantification

Blood samples were centrifuged (3500 rpm/10 min) for plasma collection. Quantification of anti-DNA antibodies was performed by indirect immunofluorescence using NOVA Lite® dsDNA Crithidia luciliae kit (Inova Diagnostics, USA) following manufacturer protocol. Goat anti-Mouse IgG (H + L) Cross-Adsorbed Secondary Antibody, Alexa Fluor 488 (Invitrogen, A-11001) was utilized at 1:100 for conjugation.

### Histological analyses of kidneys

After euthanasia, kidneys were stored in buffered formaldehyde (10%) for 24hs. Then, kidneys were processed and embedded in paraffin. For stereological analysis kidneys were sliced (5 μm thick) in serial sections spaced by 100 μm and stained with hematoxylin-eosin (HE). In the interval between serial sections, sets of sections were collected for immunohistochemistry and histopathology. HE slides were scanned and digitalized using Pannoramic SCAN (3DHistech, Hungary). Volume estimation of kidney compartments was determined using Cavalieri method [35]. Using ImageJ software (Media Cybernetics, USA), a grid was superposed over the digitalized images (× 10 magnification) and the incident points were counted. Relative volumes of cortex and medulla were calculated by dividing the sum of incident points of each compartment by the sum of total points of the kidney. Absolute volumes of compartments were calculated by multiplying relative volumes by total kidney volume. Total kidney volume was estimated by dividing kidney weight by kidney density (d = 1,06 g/cm^3^).

For histopathologic analysis kidneys slices were randomly selected and stained using Periodic acid-Schiff (PAS). Slide sections were individually graded by a board-certified veterinary pathologist using a modified International Society of Nephrology-Renal Pathology Society Lupus Nephritis Classification [36] as follows: (0) no tubular proteinosis and normal glomeruli; (1) mild tubular proteinosis with multifocal segmental proliferative glomerulonephritis and occasional early glomerular sclerosis and crescent formation; (2) moderate tubular proteinosis with diffuse segmental proliferative glomerulonephritis, early glomerular sclerosis and crescent formation; and (3) marked tubular proteinosis with diffuse global proliferative and sclerosing glomerulonephritis. Glomerular volume was estimated by measuring average diameter of glomeruli (× 400 magnification) [37].

For analysis of collagen deposition another set of randomly selected were stained by picrosirius-hematoxylin method [38]. Slides were scanned and digitalized as previously described. Ten pictures per animal were taken. Threshold tool of ImageJ software (Media Cybernetics, USA) was utilized for estimation of the area stained with picrosirius (× 100 magnification).

### Immunohistochemistry

Immunohistochemistry protocol followed the manufacturer instructions. Briefly, sections were hydrated, antigen retrieval was performed in sodium citrate buffer (pH = 6) at 96 °C/20 min, endogen peroxidase was blocked with methanol (95%) + peroxide hydrogen (5%) for 30 min and non-specific bindings were blocked with bovine serum albumin (2%) for 60 min. Then we incubated sections with primary antibodies anti-C3 (1:2000 - ab200999), anti-IgG (1:250 - ab190475) and macrophage marker (1:10 - sc-101447) at 4 °C/overnight. Next, slices were incubated with HRP-polymer anti-rabbit (ab214880) or HRP-polymer anti-rat (ab214882) for 90 min and the chromogen 3,3′-diaminobenzidine (DAB) was added. Counterstaining was performed with hematoxylin. Slides were scanned and digitalized as described previously. Ten pictures per animal were taken. Threshold tool of ImageJ software (Media Cybernetics, USA) was utilized for estimation of area stained with DAB (× 100 magnification).

### Gene expression

Total RNA from kidneys was extracted with Trizol reagent (Invitrogen, USA). Kidneys were immersed in trizol and macerated in Precellys (Bertin Instruments, France). Chloroform was added, the homogenate was centrifuged, and the supernatant (RNA) was collected. RNA precipitation was performed with isopropanol. After centrifugation RNA pellet was washed with ethanol 70% twice and diluted in DEPC H_2_O. Quantification of total RNA (ng/μL) and quality of extraction (260/230) was performed using Nanodrop (Thermo Scientific, USA). Synthesis of cDNA was performed with 2000 ng of total RNA. First step was the degradation of contaminant DNA with RNAase-free DNase (Promega, USA). Second step was the addition of oligo-dT (Promega, USA). Last step was the reaction with dNTP and M-MLV Reverse Transcriptase (Promega, USA). All steps were performed in Mastercycler (Eppendorf, Germany). Relative gene expression of NFκB and TGF-β was determined by quantitative real-time PCR following 2^-ΔΔCT^ method [39]. HPRT was utilized as endogenous gene (Additional file 2). For each gene, 4 μL cDNA sample + 0,5 μL forward primer + 0,5 μL reverse primer + 5 μL mastermix SYBR (Invitrogen, USA) were assembled. Samples were run in duplicates. qRT-PCR was performed at Quantstudio 12 K Flex (Applied Biosystems, USA).

### Oxidative stress

Pro-oxidant (NADPH oxidase and hydrogen peroxide) and antioxidant (FRAP) assays were performed for oxidative stress analysis of kidneys following procedures described elsewhere [40]. Firstly, kidneys were homogenized in an Ultra80 Turrax Blender (UltraStirrer, Switzerland) with 120 mM KCl and 20 nM sodium phosphate buffer (1 g tissue/4 mL solution) and centrifuged at 600 rpm/10 min. Activity of NADPH oxidase enzyme was determined by ELISA based on superoxide anion production [41]. The assay was performed with 50 mM phosphate buffer containing 2 mM EDTA and 150 mM sucrose, 1.3 mM NADPH and 10 μL of homogenate samples. Superoxide production was determined using a spectrometer (SpectraMax2, Molecular Devices) at 340 nm wavelength. Hydrogen peroxide quantification was determined by oxidation of phenol red by radish peroxidase [42]. 70 μL of homogenate samples were added in 180 μL of radish peroxidase solution (dextrose buffer + phenol red + radish peroxidase type II). After 25 min of incubation, 5 μL of sodium hydroxide were added to stop the reaction and absorbance was determined in a spectrometer (SpectraMax2, Molecular Devices) at 630 nm wavelength. Non-enzymatic antioxidant activity was determined by ferric reducing antioxidant power (FRAP) [43]. 10 μL of homogenate samples were added in 290 μL of FRAP reactive (sodium acetate and acetic acid buffer + 10 mM TPTZ + 20 mM hexahydrate ferric chloride). After 5 min of incubation, absorbance was determined in a spectrometer (SpectraMax2, Molecular Devices) at 593 nm wavelength.

### Statistical analyses

SPSS software version 17.0 (IBM, USA) was utilized for statistical analyses. Firstly, descriptive data were obtained, and normality was verified by Kolmogorov-Smirnov test. Variables with normal distribution were analyzed through the parametric test ANOVA and post-hoc tests of Tukey HSD, Gabriel or Games Howell. Test-T was performed for comparison between 2 groups. Variables with non-normal distribution were ranked and analyzed through ANOVA. Kruskal-Wallis (4 groups) and Mann-Whitey test (2 groups) were also applied for non-normal parameters. Survival analysis was performed with Kepler-Maier test. Differences between groups were considered statistically significant when *p* value was less than 0.05 (*p* < 0.05).

## Results

### PM2.5 characterization

Elemental composition of ambient PM2.5 is presented in Table 1. Characterization of main pollutants and climatic conditions are shown in Additional file 3.

**Table 1:** Elemental composition of PM2.5

Element	%
Carbon (C)	52.24 ± 6.24
Sulphur (S)	33.59 ± 2.11
Magnesium (Mg)	10.19 ± 8.56
Barium (Ba)	1.34 ± 0.47
Chloride (Cl)	1.00 ± 0.28
Phosphorus (P)	0.73 ± 0.42
Iron (Fe)	0.34 ± 0.13
Calcium (Ca)	0.28 ± 0.09
Potassium (K)	0.15 ± 0.03
Silicon (Si)	0.04 ± 0.07

Bars represent percentage mean ± error. *n* = 20 filters

### Survival analysis

**Fig. 2:**
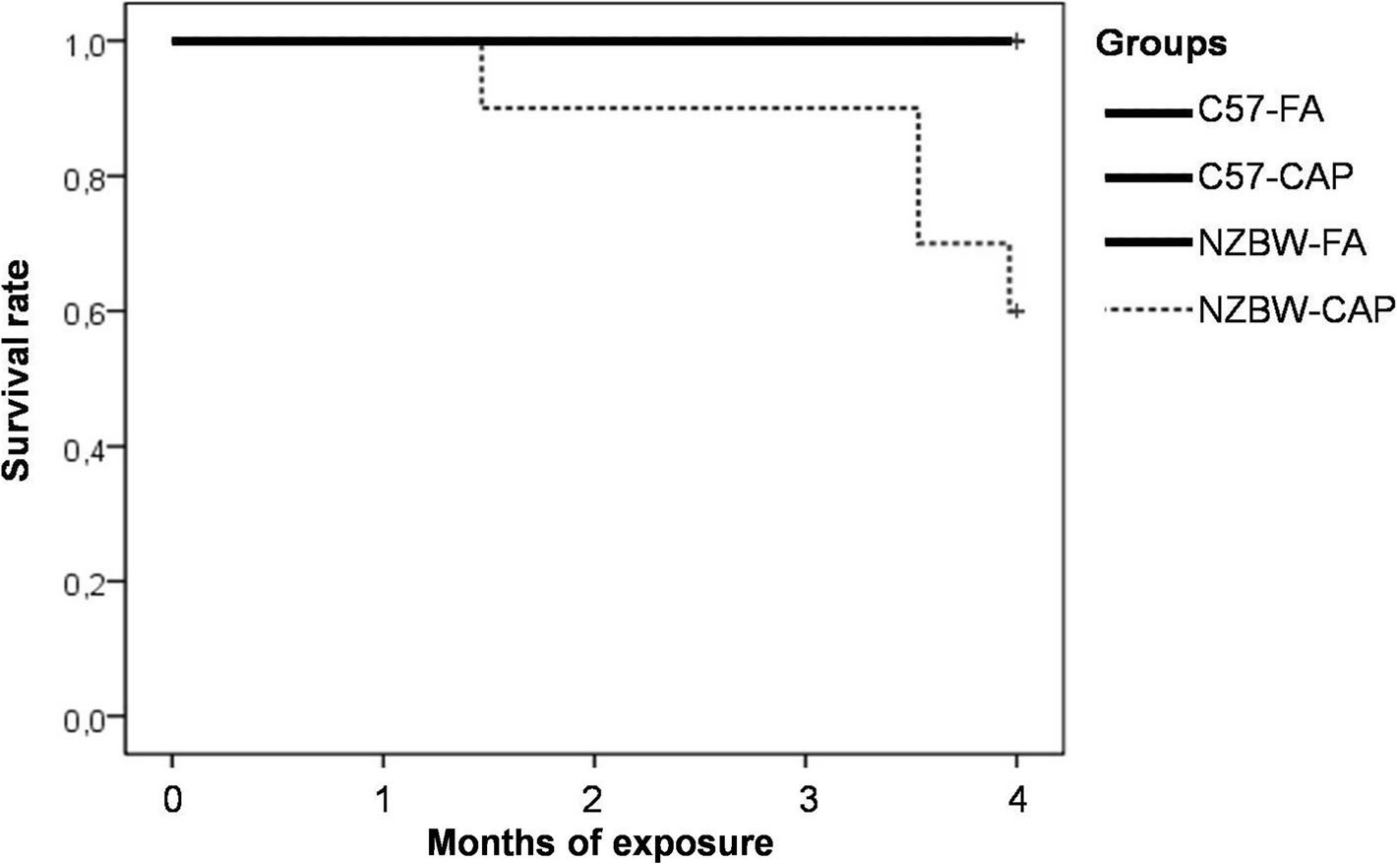
Survival rate during the period of exposure to FA or CAP. *n* = 8–10 animals/group. *p* = 0.007 (Kepler-Meier test). C57-FA: control-filtered; C57-CAP: control-pollution; NZBW-FA: lupus-filtered; NZBW-CAP: lupus-pollution

Four animals from NZBW-CAP died before the euthanasia procedure. Necropsy of the animals indicated that they died of renal insufficiency due to glomerulonephritis. No deaths occurred on C57-FA, C57-CAP and NZBW-FA groups (p = 0.007) (Fig. 2).

### Body weight

**Fig. 3:**
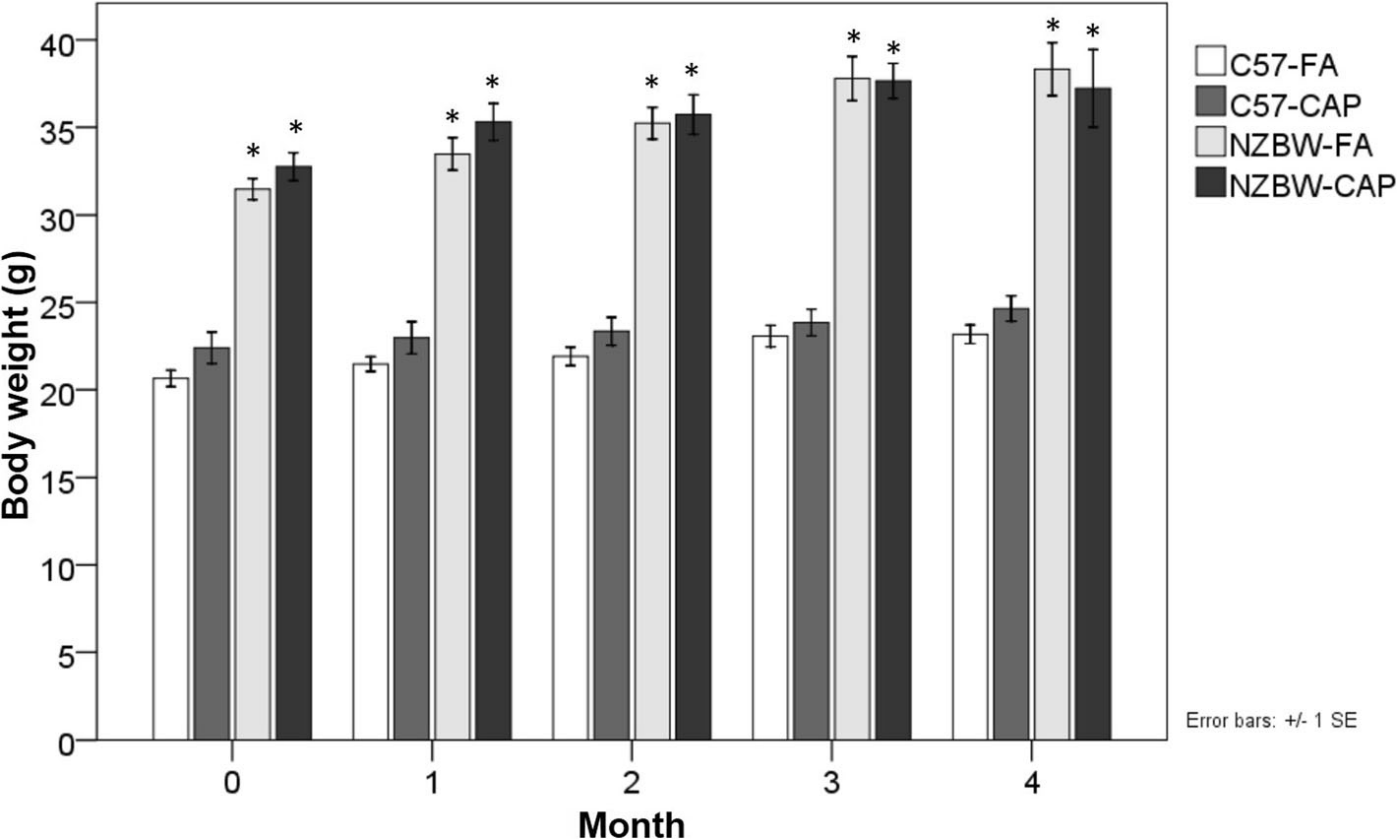
Body weight (g) during the period of exposure to FA or CAP. Bars represent mean ± standard error. n = 8–10 animals/group. *p < 0.01 when compared to both C57-FA and C57-CAP (ANOVA followed by post-hoc test Gabriel or Games-Howell). C57-FA: control-filtered; C57-CAP: control-pollution; NZBW-FA: lupus-filtered; NZBW-CAP: lupus-pollution

Both NZBW-FA and NZBW-CAP groups showed a higher body weight when compared to both C57-FA and C57-CAP groups during the whole period (*p* < 0.010). However, comparing animals of same strain, there were no differences between groups exposed to FA or CAP (Fig. 3).

### Weight of organs

In regarding to absolute weight of organs, NZBW-FA and NZBW-CAP groups showed increased values when compared to C57-FA and C57-CAP groups for kidney (*p* < 0.001), liver (*p* < 0.001) and heart (p < 0.001). Kidney weight was higher in NZBW-CAP when compared to NZBW-FA (*p* = 0.025). Spleen weight was higher in NZBW-CAP when compared to C57-FA (*p* = 0.027) and NZBW-FA (*p* = 0.046). Thymus weight was higher in NZBW-CAP and C57-FA groups when compared to C57-CAP (*p* = 0.006; *p* = 0.011) and NZBW-FA (*p* = 0.006; *p* = 0.011) (Table 2). NZBW-FA group showed lower relative weight of all organs (kidney (*p* < 0.001), spleen (*p* = 0.006), liver (*p* < 0.001), heart (*p* = 0.042) and thymus (*p* < 0.001)) when compared to C57-FA group. Except for the liver (*p* = 0.052) and the heart (*p* = 0.099), NZBW-CAP group showed higher relative weights of organs when compared to NZBW-FA group (kidney (*p* < 0.001), spleen (*p* = 0.029) and thymus (*p* = 0.007)) (Table 2).

**Table 2:** Weight of organs (absolute and relative)

		C57-FA	C57-CAP	NZBW-FA	NZBW-CAP
Absolute (g)	Kidney	0.153 ± 0.008	0.162 ± 0.024	0.203 ± 0.014^a,b^	0.263 ± 0.040^a,b,c^
	Spleen	0.082 ± 0.008	0.084 ± 0.015	0.094 ± 0.027	0.173 ± 0.124^a,c’^
	Liver	1.127 ± 0.107	1.080 ± 0.197	1.549 ± 0.137^a,b^	1.807 ± 0.488^a,b^
	Heart	0.104 ± 0.004	0.120 ± 0.015^a’^	0.157 ± 0.014^a,b^	0.177 ± 0.041^a,b^
	Thymus	0.091 ± 0.020	0.057 ± 0.019^a^	0.059 ± 0.013^a^	0.106 ± 0.045^b,c^
Relative (%)	Kidney	0.66 ± 0.05	0.66 ± 0.06	0.53 ± 0.04^a,b^	0.73 ± 0.17^c^
	Spleen	0.35 ± 0.02	0.34 ± 0.04	0.24 ± 0.07^a,b^	0.45 ± 0.26^c’^
	Liver	4.87 ± 0.39	4.36 ± 0.61^a’^	4.06 ± 0.29^a^	4.88 ± 0.94
	Heart	0.45 ± 0.03	0.49 ± 0.06	0.41 ± 0.03^a,b^	0.48 ± 0.10
	Thymus	0.39 ± 0.08	0.23 ± 0.08^a^	0.15 ± 0.03^a^	0.28 ± 0.09^a,c^

Values represented by mean ± standard deviation. *n* = 7–10 animals/group. ANOVA followed by post-hoc test Gabriel or Games-Howell or T-test

^a,a’^ *p* < 0.05 when compared to C57-FA (^a^ ANOVA; ^a’^ T-test)

^b^ p < 0.05 when compared to C57-CAP

^c,c’^ *p* < 0.05 when compared to NZBW-FA (^c^ ANOVA; ^c’^ T-test)

C57-FA: control-filtered; C57-CAP: control-pollution; NZBW-FA: lupus-filtered; NZBW-CAP: lupus-pollution

### Detection of anti-DNA antibodies

Semi-quantitative analysis of anti-DNA showed that only NZBW animals had a positive reaction. NZBW-FA showed 10% of positivity and NZBW-CAP showed 14.29%. However, there were no statistical differences between groups.

### Hemogram

There were no differences in the number of erythrocytes between groups. NZBW-FA group showed a decreased number of total leukocytes (*p* = 0.018) and lymphocytes (*p* = 0.006) when compared to C57-FA group. NZBW-CAP group showed an increased number of neutrophils (*p* = 0.017) when compared to NZBW-FA group. Also, when compared to all other groups, NZBW-CAP showed a decreased percentage of lymphocytes (C57-FA (*p* < 0.001), C57-CAP (*p* = 0.002), NZBW-FA (*p* = 0.018)), and an increased percentage of neutrophils (C57-FA (*p* = 0.001), C57-CAP (*p* = 0.002), NZBW-FA (*p* = 0.016)). The number of platelets was lower in NZBW-FA group when compared to C57-CAP group (*p* = 0.037) (Table 3).

**Table 3:** Hemogram analysis of erythrocytes, platelets and leukocytes (total, neutrophils, lymphocytes and monocytes)

	C57-FA	C57-CAP	NZBW-FA	NZBW-CAP
Erythrocytes^1^	9.43 ± 0.30	8.71 ± 0.62	10.39 ± 1.89	7.90 ± 2.03
Platelets^2^	1119.2 ± 115.8	1334.3 ± 396.6	664.8 ± 368.2^b^	684.0 ± 307.0
Leukocytes^2^	3.34 ± 0.96	2.70 ± 0.59	1.72 ± 0.51^a^	2.70 ± 0.66
Lymphocytes^2^	2.54 ± 0.74	2.00 ± 0.32	1.20 ± 0.40^a^	1.50 ± 0.31
Neutrophils^2^	0.71 ± 0.24	0.67 ± 0.28	0.50 ± 0.15	1.13 ± 0.31^c^
Monocytes^2^	0.09 ± 0.08	0.03 ± 0.01	0.02 ± 0.01	0.06 ± 0.06
Lymphocytes (%)	76.00 ± 5.05	74.75 ± 5.12	69.40 ± 5.86	56.00 ± 2.65^a,b,c^
Neutrophils (%)	21.40 ± 3.91	24.00 ± 5.35	29.40 ± 5.81	41.67 ± 1.53^a,b,c^
Monocytes (%)	2.60 ± 1.67	1.25 ± 0.50	1.20 ± 0.45	2.00 ± 1.73

Values represented by mean ± standard deviation. ^1^(million/mm^3^); ^2^(thousand/mm^3^). *n* = 3–5 animals/group. ANOVA followed by post-hoc test Gabriel

^a^ *p* < 0.05 when compared to C57-FA

^b^ p < 0.05 when compared to C57-CAP

^c^ p < 0.05 when compared to NZBW-FA

C57-FA: control-filtered; C57-CAP: control-pollution; NZBW-FA: lupus-filtered; NZBW-CAP: lupus-pollution

### Proteinuria

**Fig. 4:**
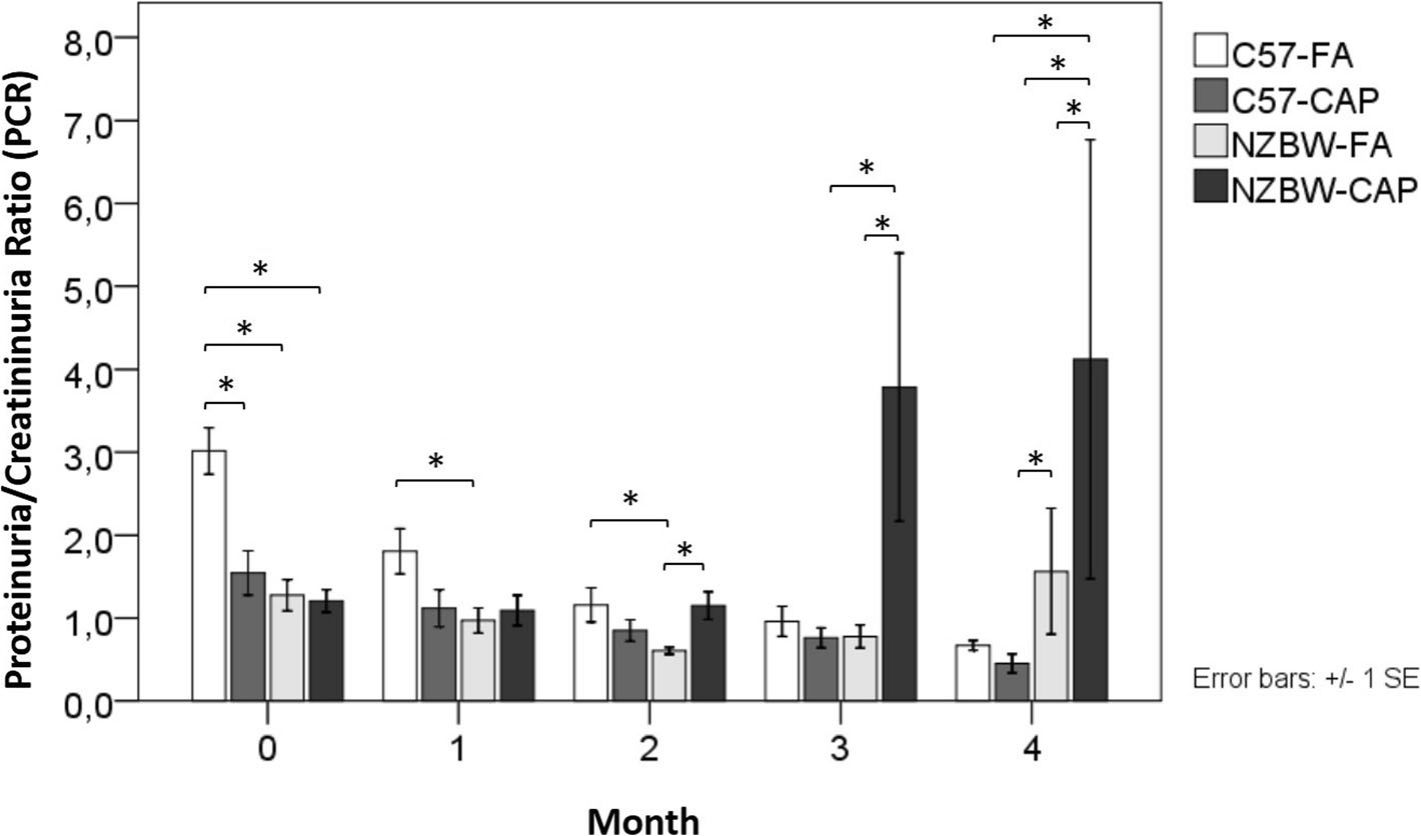
Proteinuria/creatininuria ratio (PCR) during the period of exposure to FA or CAP. Bars represent mean ± standard error. *n* = 5–10 animals/group. *p < 0.05 (ANOVA followed by post-hoc test Gabriel). C57-FA: control-filtered; C57-CAP: control-pollution; NZBW-FA: lupus-filtered; NZBW-CAP: lupus-pollution

PCR was followed during the whole period of exposure (from month 0 to month 4). At month 0, NZBW-FA (*p* < 0.001), NZBW-CAP (p < 0.001) and C57-CAP (*p* = 0.002) showed decreased PCR when compared to C57-FA. NZBW-FA maintained a lower PCR when compared to C57-FA at month 1 (*p* = 0.045) and month 2 (*p* = 0.003). NZBW-CAP showed increased PCR when compared to NZBW-FA at month 2 (*p* = 0.001), month 3 (*p* = 0.006) and month 4 (*p* = 0.034). NZBW-CAP also showed increased PCR when compared to C57-CAP at month 3 (*p* = 0.046) and month 4 (p < 0.001). At month 4, NZBW-FA showed higher PCR when compared to C57-CAP (*p* = 0.021), and NZBW-CAP showed increased PCR when compared to C57-FA (*p* = 0.001) (Fig. 4). ACR was performed with urine collected at the day of euthanasia. There were no differences between the groups.

### Stereology of kidneys

**Fig. 5:**
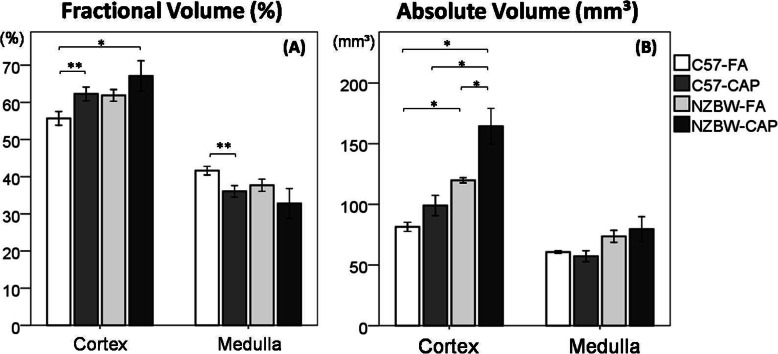
Stereological analysis of kidneys for estimation of fractional (**a**) and absolute (**b**) volumes of kidney compartments. Bars represent mean ± standard error. n = 5–6 animals/group. *p < 0.05 (ANOVA followed by post-hoc test Gabriel or Games-Howell). C57-FA: control-filtered; C57-CAP: control-pollution; NZBW-FA: lupus-filtered; NZBW-CAP: lupus-pollution

Fractional volume of kidney major compartments estimated by Cavalieri principle showed that C57-CAP (*p* = 0.035) and NZBW-CAP (*p* = 0.032) groups showed an increase in the volume of cortex when compared to C57-FA group, and C57-CAP showed a decrease in the volume of medulla when compare to C57-FA (p = 0.021) (Fig. 5a). In absolute volume analysis, NZBW-FA group showed a larger volume of cortex when compared to C57-FA (*p* = 0.001) and NZBW-CAP group showed a larger volume of cortex when compared to all the other groups (C57-FA (*p* < 0.001); C57-CAP (p < 0.001); NZBW-FA (*p* = 0.034)) (Fig. 5b).

### Histopathology of kidneys

**Fig. 6:**
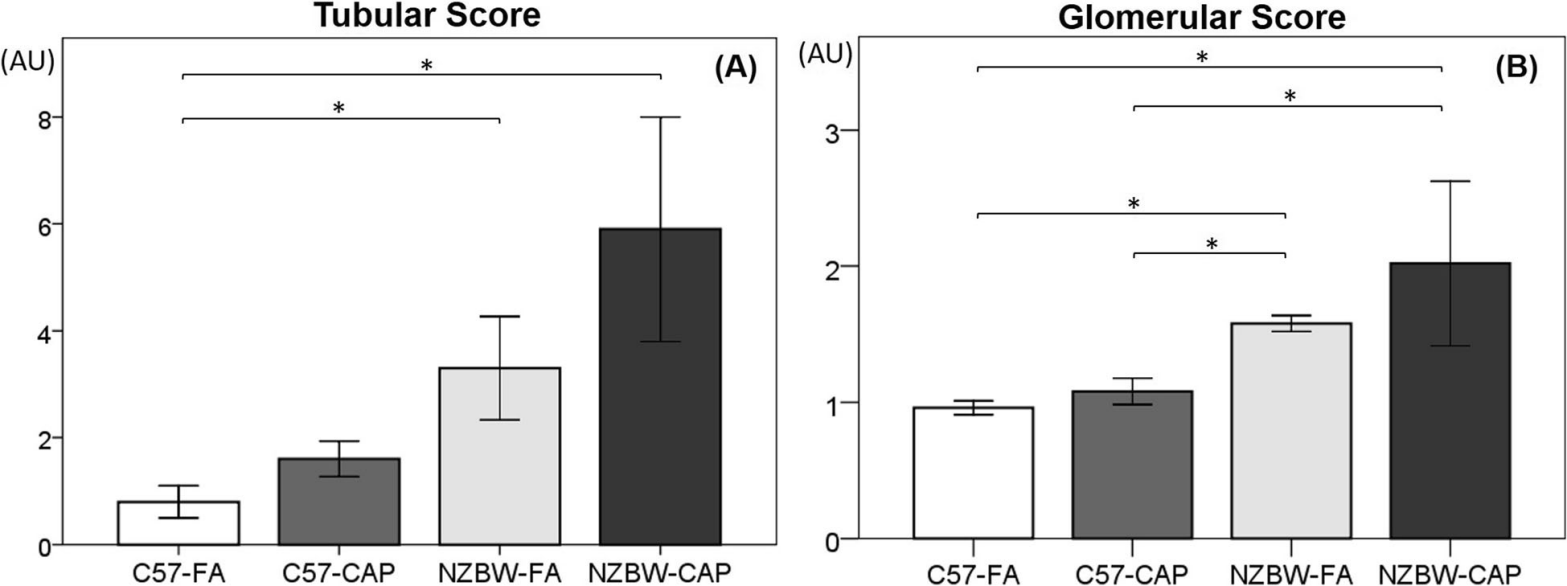
Kidney histopathology (arbitrary units) of tubules (**a**) and glomeruli (**b**). Bars represent mean ± standard error. n = 5 animals/group. *p < 0.05 (ANOVA followed by post-hoc test Tukey HSD). C57-FA: control-filtered; C57-CAP: control-pollution; NZBW-FA: lupus-filtered; NZBW-CAP: lupus-pollution

In histopathological analysis of the renal tubules, both NZBW-FA (*p* = 0.044) and NZBW-CAP (*p* = 0.005) groups showed a higher score when compared to C57-FA (Fig. 6a). Glomerular evaluation indicated a higher score in NZBW-CAP when compared to C57-FA (*p* = 0.003) and C57-CAP (*p* = 0.025). Also, NZBW-FA showed a higher score when compared to C57-FA (p = 0.001) and C57-CAP (*p* = 0.010) (Fig. 6b). A summary description of the histopathological findings of kidneys together with stereological results for each group is described in Additional file 4. No differences between the groups were observed regarding the mean volume of glomeruli.

### Picrosirius analysis

There were no differences between the groups in the fractional area positively stained with picrosirius, indicating fibrosis.

### Immunohistochemistry

**Fig. 7:**
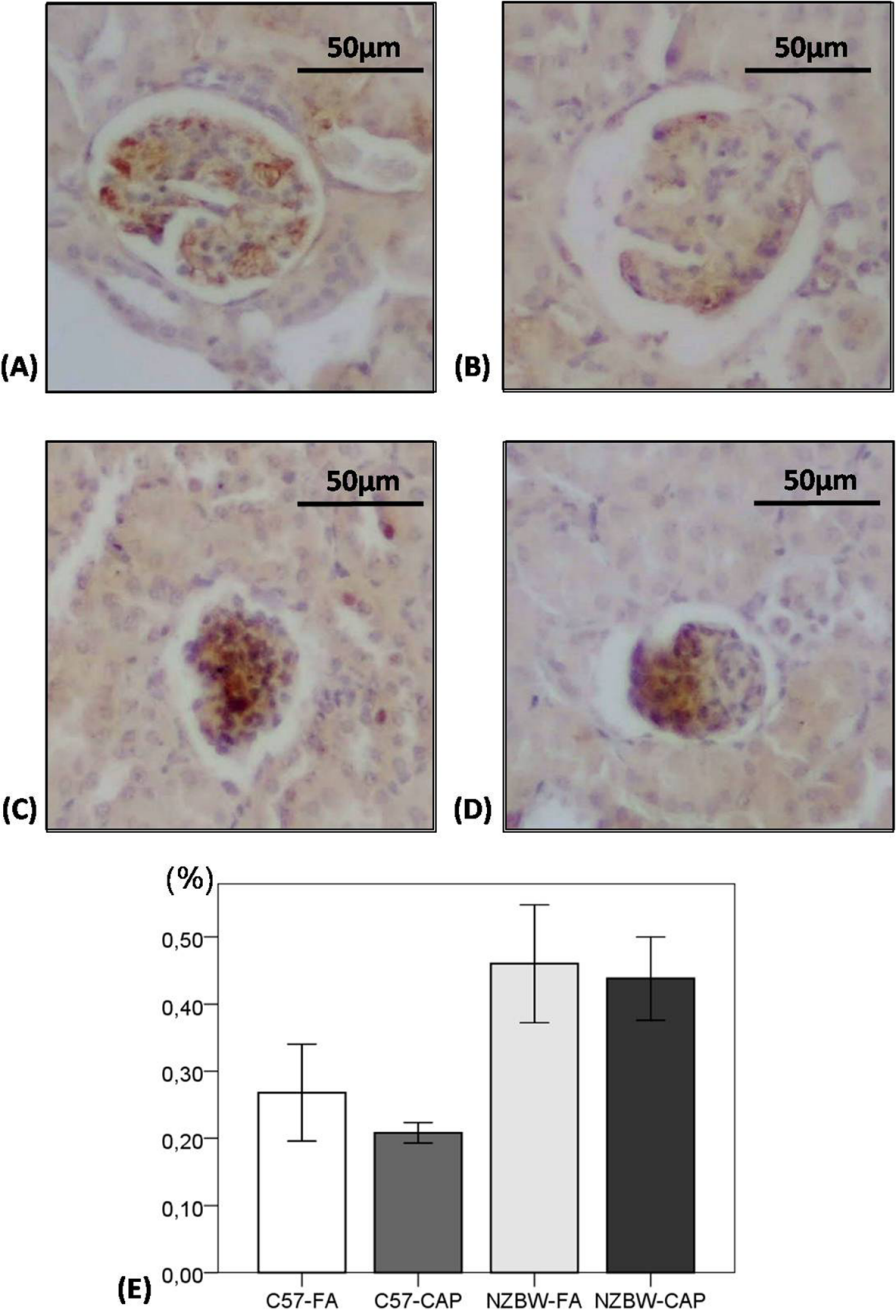
Photomicrographs of kidneys from C57-FA (**a**), C57-CAP (**b**), NZBW-FA (**c**) and NZBW-CAP (**d**) groups for quantification of IgG deposition (**e**) by IHC. Bars represent mean ± standard error. n = 5–7 animals/group. ANOVA followed by post-hoc test Games-Howell. C57-FA: control-filtered; C57-CAP: control-pollution; NZBW-FA: lupus-filtered; NZBW-CAP: lupus-pollution

**Fig. 8:**
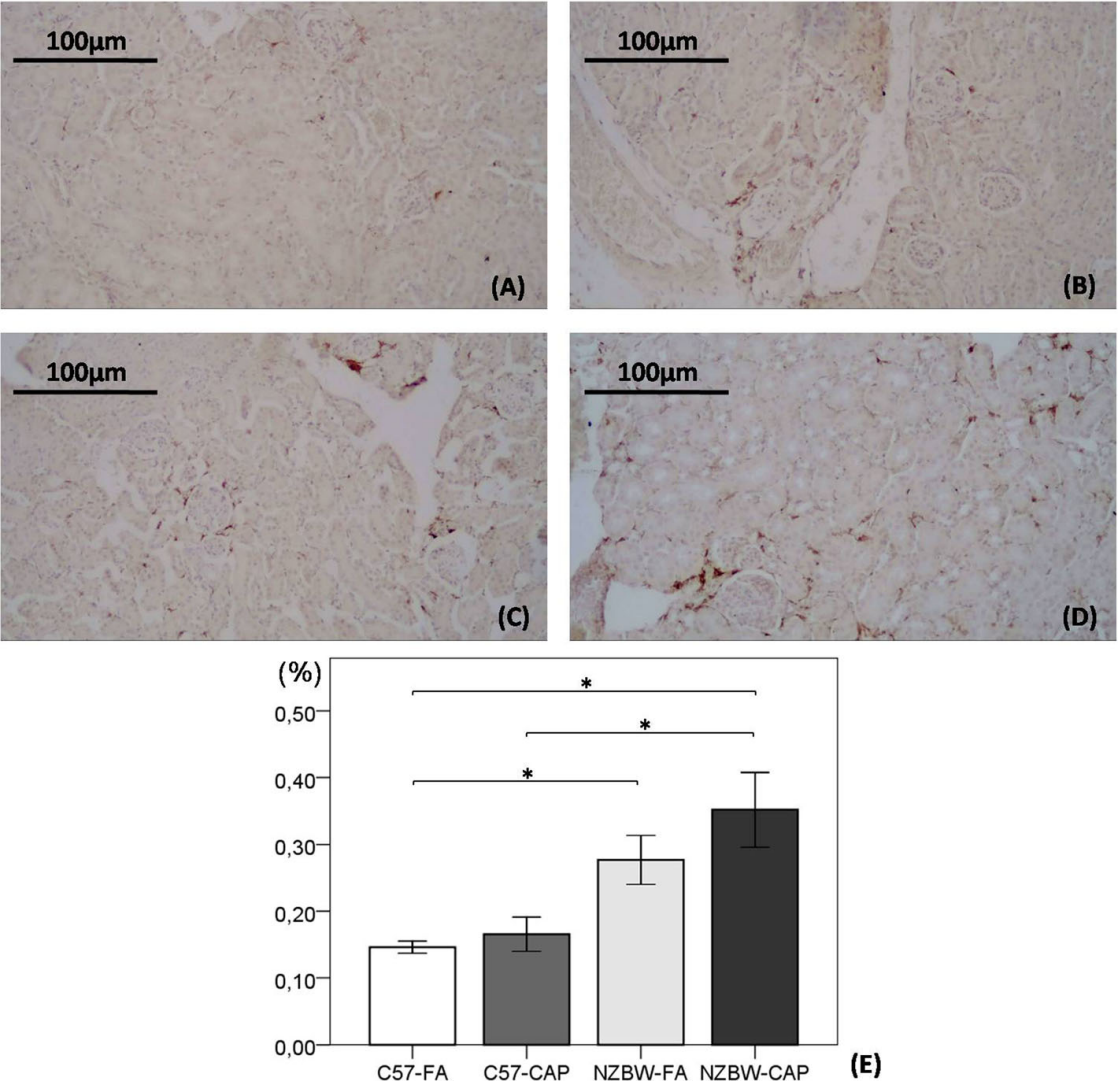
Photomicrographs of kidneys from C57-FA (**a**), C57-CAP (**b**), NZBW-FA (**c**) and NZBW-CAP (**d**) groups for quantification of macrophages (**e**) by IHC. Bars represent mean ± standard error. n = 5–8 animals/group. *p < 0.05 (ANOVA followed by post-hoc test Gabriel). C57-FA: control-filtered; C57-CAP: control-pollution; NZBW-FA: lupus-filtered; NZBW-CAP: lupus-pollution

Deposition of C3 complement positively marked with DAB showed no differences between the groups. We observed a tendency to increased deposition of IgG in NZBW mice, without differences between NZBW-FA and NZBW-CAP groups (Fig. 7). Macrophage quantification was significantly higher in NZBW-FA (*p* = 0.023) and NZBW-CAP (*p* = 0.007) groups in comparison to C57-FA group and, NZBW-CAP showed an increase when compared to C57-CAP group (*p* = 0.028). However, no differences were observed between NZBW-FA and NZBW-CAP groups (Fig. 8).

### Gene expression

**Fig. 9:**
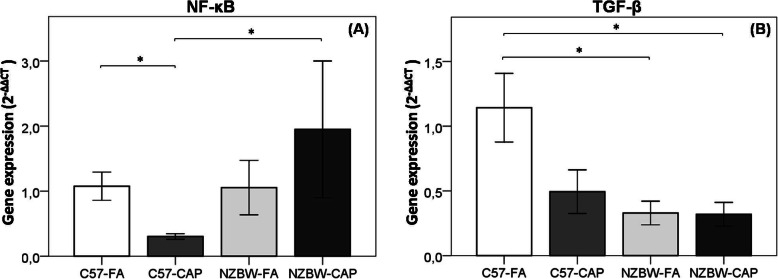
Analysis of gene expression of NF-κB (**a**) and TGF-β (**b**) by qRT-PCR. (2^-ΔΔCT^ method). Bars represent mean ± standard error. n = 5–10 animals/group. *p < 0.05 (ANOVA followed by post-hoc test Gabriel). C57-FA: control-filtered; C57-CAP: control-pollution; NZBW-FA: lupus-filtered; NZBW-CAP: lupus-pollution

Gene expression of NF-κB was lower in C57-CAP group when compared to C57-FA (*p* = 0.010) and NZBW-CAP (*p* = 0.032) groups (Fig. 9a). TGF-β expression was higher in C57-FA when compared to NZBW-FA (*p* = 0.002) and NZBW-CAP (*p* = 0.005) groups (Fig. 9b). No significant differences were observed between NZBW-FA and NZBW-CAP groups.

### Oxidative stress

**Fig. 10:**
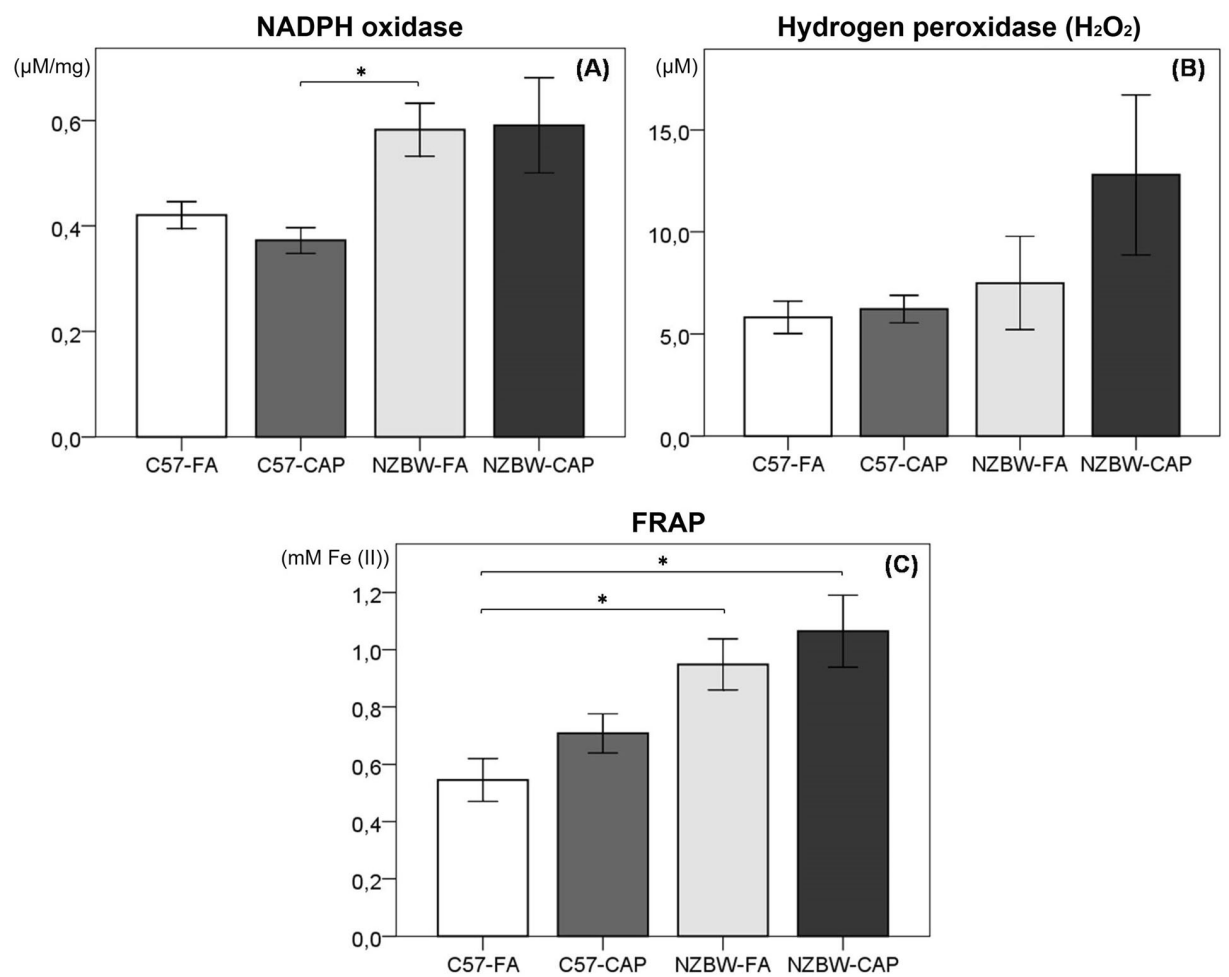
Quantification of NADPH oxidase (μM/mg protein) (**a**), hydrogen peroxide (μM) (**b**) and ferric reducing antioxidant activity (FRAP) (mM Fe (II)) (**c**) in kidneys. Bars represent mean ± standard error. n = 8–10 animals/group. *p < 0.05 (ANOVA followed by post-hoc test Gabriel or Games-Howell). C57-FA: control-filtered; C57-CAP: control-pollution; NZBW-FA: lupus-filtered; NZBW-CAP: lupus-pollution

NADPH oxidase activity was higher in group NZBW-FA when compared to C57-CAP (*p* = 0.011) and a tendency of increase was observed when compared to C57-FA (*p* = 0.055) (Fig. 10a). Quantification of hydrogen peroxide showed no differences between groups (Fig. 10b). FRAP activity was higher in NZBW-FA (*p* = 0.048) and NZBW-CAP (*p* = 0.021) when compared to the control group (C57-FA) (Fig. 10c). No differences were observed between NZBW-FA and NZBW-CAP groups in oxidative stress analyses.

## Discussion

Chronic exposure to PM 2.5 leads to the exacerbation of some clinical and renal histopathological manifestations of SLE in a murine model of lupus disease (NZBWF1 mice). Different outcomes were evaluated, however the most striking result is the decreased survival rate in lupus prone mice exposed to particulate air pollution and marked changes in renal structure (increases in kidney weight and renal cortex volume) and function (early proteinuria onset), as well as the elevated number of circulating neutrophils.

Autoimmune diseases are very complex diseases and can affect different organs due to loss of self-tolerance and inappropriate activation of the immune system that leads to the production of autoantibodies and generates tissue destruction. The most common autoimmune diseases are: SLE, rheumatoid arthritis, multiple sclerosis and type 1 diabetes [10]. The etiology of these diseases is still under investigation, however, besides a genetic predisposition, environmental factors (e.g., urban air pollution) seems to contribute to the onset and worsening of these diseases manifestations [8].

Recent epidemiological studies clearly show an association between air pollution and disease activity in patients with SLE, but there is no agreement of which of the pollutants present in the air are most associated to the outcomes. Possibly, the differences between studies occur because the disease is strongly related to race, gender and age [44–47]. In vitro studies showed that PM can induce T lymphocyte activation, thus contributing to the aggravation of autoimmune diseases [48, 49]. Despite the methodological differences between epidemiological and in vitro studies, the results of these studies corroborate our findings.

As far as we know, this is the first study that investigated the effects of exposure to “real world” urban particulate air pollution (whole-body inhalation) on lupus-prone animals, a model that mimics some clinical manifestations of SLE in humans. NZBW mice were chosen as SLE animal model based on literature descriptions, since it is considered as the classic model of SLE [50, 51]. Furthermore, only females were utilized in order to mimic disease prevalence in humans (90% of adult patients are women) [2]. Female C57BL/6 was chosen as a control for the NZBW because this strain was already used as a control for NZBW in previous studies [52], and because is recommended for toxicological studies and has no predisposition to renal diseases [53].

The onset of SLE manifestations in female NZBW mice occurs around 28-week-old (7-month-old) and average lifespan is 245 days [54]. We planned our experimental design following this lifespan. Thus, exposures started 4 months before the expected onset of the clinical manifestations, in order to check if air pollution could anticipate the symptoms. Although not expected, lupus-prone mice presented a significant reduction in life expectancy (less 25%) due to exposure to air pollution and necropsy indicated kidney failure as the cause of death. Globally, different studies have already shown that air pollution leads to reductions in life expectancy [55–57]. However, to date there is no data on mortality risk for SLE patients.

Previous studies using a different mice model for SLE (New Zealand Mixed, NZM), have shown that instillation of silica particles increased mortality due to renal complications [58] and instillation of particulate matter (PM1648 and PM2.5, dose 500 μg) also increased mortality of NZM, but with absence of renal impairments [59]. In our model, NZBW mice exposed to CAP presented early development of proteinuria and the weight of the kidney was increased compared to NZBW exposed to FA, although histopathological scores, fibrosis and C3 complement deposition on kidneys were not different between these groups.

Detection of anti-DNA in serologic tests is important for confirmation of SLE diagnosis [60]. Usually, experimental studies perform anti-DNA quantification assays for evaluation of disease activity [61, 62]. As observed in previous studies with SLE-prone mice exposed to silica [52, 58, 63], we had expected an increase in detection of anti-DNA antibodies in NZBW group exposed to PM2.5. However, there were no differences between the groups. Two aspects can explain this difference: evaluation at the end of the experiment and not during the course and the low number of animals assessed. Anti-DNA antibodies participate in pathogenesis of renal lesions, but its relationship with disease activity and severity varies. Goulart and colleagues showed association between anti-DNA and renal impairments in children [47]. Other studies failed to show a clear correlation between circulating anti-DNA and the type or severity of renal disease in individual patients [64, 65].

In general, SLE patients are prone to the development of hematologic alterations such as anemia, leucopenia and thrombocytopenia, associated with symptoms such as fatigue and susceptibility to infections [66]. In the current study, we observed that NZBW-FA group presented leucopenia and thrombocytopenia, following conditions observed in human patients [60]. We did not observe differences between FA and CAP groups in erythrocytes and platelets counts; however, leukocytes were altered. Female NZBW mice exposed to CAP presented increased percentage and total number of neutrophils and decreased percentage of lymphocytes when compared to NZBW-FA group. Interestingly, a recent study demonstrated that an increased neutrophil/lymphocyte ratio in blood can be associated with increased SLE activity in human patients [67]. In our study we showed that exposure to PM2.5 alters the blood cell composition of female NZBW mice (leading to increased neutrophil/lymphocyte ratio). This result corroborates the general exacerbation of SLE manifestations in these mice.

Exposure to PM2.5 also affected the weight of organs on SLE-prone mice. Body weight did not vary, however NZBW mice exposed to PM2.5 presented higher weight of organs compared to NZBW-FA group. Increase in weight of lymphoid organs such as thymus and spleen can be associated with systemic condition of increased disease activity, characterized by hyperplasia of lymphoid follicles present in these organs [68, 69]. Increase in weight of liver is frequently associated with splenomegaly in SLE patients, however the mechanisms involved are not elucidated [70]. Increased renal weight is associated with a worse renal histopathology [71]. SLE inflammation processes caused by deposition of immune complexes, cell infiltration and formation of lymphoid structures in hepatic and renal parenchyma [72, 73] can be associated with these outcomes.

Morphometric evaluation of the kidney structure revealed that in NZBW mice exposed to CAP, the renal cortex volume was increased compared to FA group. These changes were accompanied by altered kidney function (augmented proteinuria). Increase of renal cortex can be related to hypertrophy of nephrons, characterized by large glomeruli and dilated tubules [74]. This condition is associated with increased glomerular filtration rate and, frequently, proteinuria [74, 75]. Nephron hypertrophy can occur by compensatory mechanisms when nephrons are lost due to disease progression; however, this condition impairs glomerular function over time [76, 77]. NZBW descriptions show the appearance of proteinuria around 5 to 7 months-age in females [54]. In this current study, NZBW animals exposed to CAP presented an earlier onset of proteinuria when compared to NZBW-FA group, with progression starting at month 2 (5-month-age) and intensified at month 3 (6-month-age) and month 4 (7-month-age). Although proteinuria appeared early on NZBW exposed to CAP, we did not observe a significant increase in the mean volume of glomeruli. Whether the increase of renal cortex can be influenced by interstitial enlargement remains to be determined.

Deposition of collagen and complement proteins, markers of fibrosis and immune complexes deposition on kidneys, respectively, did not differ between groups. Other lesions such as basal membrane thickening, mesangial cell expansion and inflammation, that are characteristic of kidney injury in lupus nephritis [22], were more pronounced in NZBW strain exposed to air pollution. Some epidemiological studies reported impairment of renal function on SLE patients exposed to PM2.5. Children diagnosed with SLE and inhabitants of São Paulo city presented increased proteinuria, leukocyturia and hematuria in association with an increase in PM2.5 concentrations [47]. In the same way, adult patients diagnosed with SLE presented renal complications associated with exposure to PM2.5 in the city of Montreal, Canada [78]. Furthermore, risk of nephropathies, including lupus nephritis, were associated with long term exposure to air pollutants in China [79].

Regarding animal models of SLE, no kidney alterations were observed in a study that treated NZM mice with instilled PM [59]. On the other hand, studies with silica particles demonstrated kidney impairments with development of proteinuria and renal histopathologic alterations in lupus-prone mice instilled with silica [52, 58].

Our results corroborate the epidemiological evidence that indicates that the levels of air pollutants can increase renal activity in SLE patience [47, 78]. Furthermore, the histopathological findings in C57-CAP group indicates that even for normal individuals chronic exposure can exert negative impacts on the kidney, giving strength to previous studies that have shown an association between kidney diseases and air pollution [80]. Despite bringing new evidence of the effects of air pollution on SLE disease, our study has some limitations: the absence of a cohort to study life span properly, the small number of animals in the NZBW groups and exposure were carried in a specific period of life (near the expected onset of the SLE manifestations in NZBW mice).

In brief, our data indicate that exposure to particulate air pollution aggravates and accelerates disease progression, renal function is compromised, however the mechanisms involved in this association are still not clear. Considering previously suggested mechanisms, we expected that oxidative stress together with an increase in the complement deposition and an increased inflammatory response would explain the association between air pollution and kidney impairments [81, 82]. However, our data did not show differences between groups in NF-κB and TGF-β expressions or in the balance of pro/antioxidant molecules in the kidney. This can be partially explained by the fact that we analyzed these parameters at the end of the study, when the disease was already in an advanced stage.

## Conclusions

Despite the variability among the NZBW strain regarding to the development of disease manifestations, negative effects of air pollution in SLE-prone mice are evident, corroborating findings from epidemiological studies. Taking together, our results show that inhalation of CAP induces alterations on clinical manifestations of female NZBW. Complementary studies are important for a better elucidation of the association between air pollution and SLE and the physiopathological mechanisms involved. Reduction of air pollution levels is needed in order to provide a better quality of life for individuals diagnosed with SLE.

## Supplementary Information

**Additional file 1.**

**Additional file 2.**

**Additional file 3.**

**Additional file 4.**

## Data Availability

All the data that support the findings of this study are available on request from the corresponding author.

## Abbreviations

ACR: Albumin/creatinine ratio
CAP: Concentrated ambient particles
DAB: 3,3′-diaminobenzidine
EDX: Energy dispersive X-ray spectroscopy
FA: Filtered air
FRAP: Ferric reducing antioxidant power
HAPC: Harvard Ambient Particle Concentrator
HE: Hematoxylin-eosin
IC: Immune complexes
PAS: Periodic acid-Schiff
PM2.5: Fine particulate matter
PCR: Protein/creatinine ratio
SLE: Systemic lupus erythematosus
WHO: World Health Organization
