# Supplementary material for: Chronic exposure to PM2.5 aggravates SLE manifestations in lupus-prone mice
Source: Part Fibre Toxicol. 2021 Mar 25;18:15. doi: 10.1186/s12989-021-00407-0 (PMC7992962; doi:10.1186/s12989-021-00407-0)

**Additional file 1:** Harvard Ambient Particle Concentrator (HAPC). (A) The Exposure chambers of HAPC. (B) The three stages of virtual impactors that concentrate PM2.5. (C) Disposition of mice cages inside the exposure chambers. (D) DataRam utilized to monitor the dose of exposure.


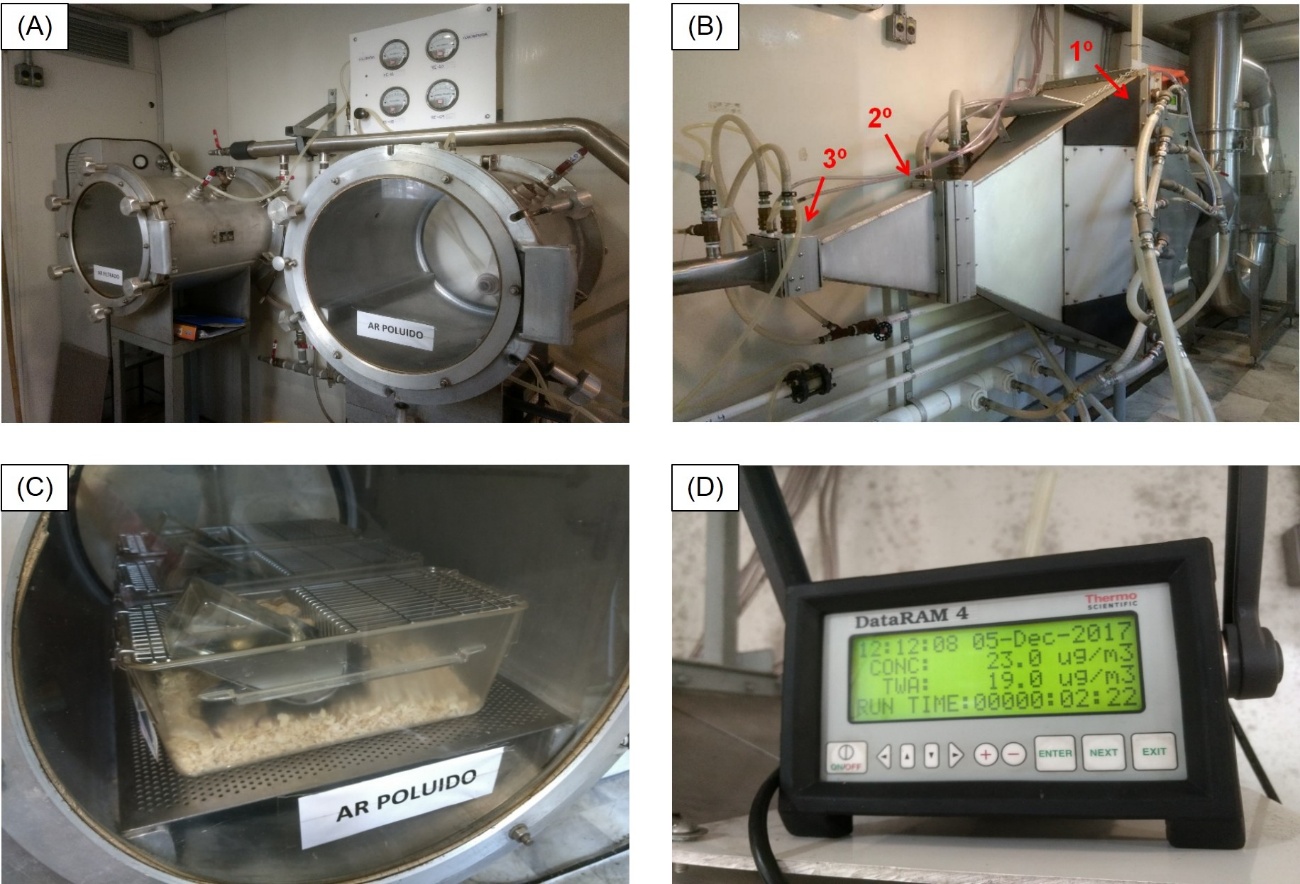

Supplement: Supplementary file 1 — Additional file 1. [file 12989_2021_407_MOESM1_ESM.docx]
