# Supplementary material for: Chronic exposure to PM2.5 aggravates SLE manifestations in lupus-prone mice
Source: Part Fibre Toxicol. 2021 Mar 25;18:15. doi: 10.1186/s12989-021-00407-0 (PMC7992962; doi:10.1186/s12989-021-00407-0)
Supplement: Supplementary file 2 — Additional file 2. [file 12989_2021_407_MOESM2_ESM.docx]

**Additional file 2:** qRT-PCR primers

| Gene | Sequence |
| --- | --- |
| *Hprt* | F: 5’ CTCATGGACTGATTATGGAC 3’  R: 5’ GCAGGTCAGCAAAGAACTTA 3’ |
| *Nfκb* | F: 5’ ATGGCAGACGATGATCCCTACGG 3’  R: 5’ ATGGGCCATCTGTTGACAGTGGT 3’ |
| *Tgfβ* | F: 5’ CTTCGACGTGACAGACGCT 3’  R: 5’ GCAGGGGCAGTGTAAACTTATT 3’ |
