# Supplementary material for: Chronic exposure to PM2.5 aggravates SLE manifestations in lupus-prone mice
Source: Part Fibre Toxicol. 2021 Mar 25;18:15. doi: 10.1186/s12989-021-00407-0 (PMC7992962; doi:10.1186/s12989-021-00407-0)
Supplement: Supplementary file 3 — Additional file 3. [file 12989_2021_407_MOESM3_ESM.docx]

**Additional file 3:** Characterization of main pollutants and climatic conditions in the ambient air of the site of exposure (São Paulo city, Brazil).

| Parameter | Mean | SD |
| --- | --- | --- |
| PM2.5 (µg/m³) | 20.37 | 8.25 |
| NO_2_ (µg/m³) | 59.67 | 1.15 |
| SO_2_ (µg/m³) | 2.33 | 0.58 |
| Temperature (ºC) | 24.00 | 1.34 |
| Relative Humidity (%) | 59.47 | 7.63 |
