# Supplementary material for: Chronic exposure to PM2.5 aggravates SLE manifestations in lupus-prone mice
Source: Part Fibre Toxicol. 2021 Mar 25;18:15. doi: 10.1186/s12989-021-00407-0 (PMC7992962; doi:10.1186/s12989-021-00407-0)
Supplement: Supplementary file 4 — Additional file 4. [file 12989_2021_407_MOESM4_ESM.docx]

**Additional file 4:** Summary of the histopathological and stereological findings of kidneys for each group

| Groups | Histopathological and stereological description - kidney |
| --- | --- |
| C57-FA | Normal and regular appearance of the kidneys by light microscopy |
| C57-CAP | Normal histological appearance of the kidneys structure by light microscopy. Detailed examination of the components of the cortex revealed that C57 mice exposed to CAP present focal thickening of the basal membrane, non-frequent expansion of the Bowman's capsule, increases in mesangial cell number and, apoptotic figures in the tubules are not rare. And although rare we can observe atrophic/sclerotic glomeruli. |
| NZBW-FA | Histopathological examination of the kidneys of NZBW-FA mice (not exposed to CAP) revealed the expected changes associated to the progression of SLE in this model. Briefly, tubular degeneration, global and segmental glomerular sclerosis, proteinaceous deposits, mesangial and endothelial cell proliferation, glomerular crescents and focal inflammatory infiltrates were observed. |
| NZBW-CAP | Macroscopically the kidneys of NZBW-CAP mice were increased (weight and volume), and stereological examination of the kidney compartments indicated that the total volume of the cortex was higher compared to NZBW-FA mice. Microscopic examination of the kidney revealed marked differences in disease progression compared to NZBW-FA group. Histopathological examination indicated that exposure to CAP accentuated the disease manifestation or its progression. We observed important vascular, glomerular, and tubulointerstitial lesions. |
